# Clarification of possible ordered distributions of trivalent cations in layered double hydroxides and an explanation for the observed variation in the lower solid-solution limit

**DOI:** 10.1107/S2052519213027905

**Published:** 2013-11-18

**Authors:** Ian G. Richardson

**Affiliations:** a School of Civil Engineering, University of Leeds, Woodhouse Lane, Leeds LS2 9JT, England

**Keywords:** layered double hydroxide, trivalent cations, supercell parameter

## Abstract

The sequence of hexagonal ordered distributions of trivalent cations that are possible in the octahedral layer of layered double hydroxides is clarified, including the link between the composition and the supercell *a* parameter. A plausible explanation is provided for the observed variation in the lower solid-solution limit.

## 1. Introduction   

Layered double hydroxide (LDH) phases are derived from layered single hydroxides [*i.e.* β-*M*(OH)_2_ phases] by the substitution of a fraction (*x*) of the divalent cations in the octahedral layer by trivalent cations. There are many natural LDH phases (Mills *et al.*, 2012 ▶) and synthetic preparations are studied widely because of their use in a wide range of applications (Cavani *et al.*, 1991 ▶). Evidence for and against long-range ordering of the trivalent cations has been discussed extensively (*e.g.* see Evans & Slade, 2006 ▶). The view of Drits & Bookin (2001 ▶) is that complete cation ordering probably depends on both the *M* ^2+^:*M* ^3+^ ratio and the conditions of crystallization, with those conditions that produce single crystals more preferable for cation order than those that produce finely dispersed material, which was considered likely to be accompanied by some heterogeneity that would result in imperfect long-range cation order. This would mean that supercell reflections would be absent or difficult to observe. The values of *x* of 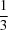 and 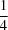 are the two largest values that are possible for hexagonal ordered distributions of trivalent cations (Brindley & Kikkawa, 1979 ▶) and as a consequence these are the most commonly studied compositions (Richardson, 2013*a* ▶). Other ordered distributions that correspond to lower values of *x* have been considered but there is dispute concerning the exact supercell parameters that are possible. For example, Drits & Bookin (2001 ▶) refer to supercell parameters of 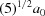 and 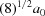, but – as will be demonstrated in this paper – neither of these is possible (*a*_0_ is the *a* parameter for the cell where there is no differentiation between cations). The purpose of this paper is twofold: firstly to demonstrate unequivocally the ordered distributions that are possible, and secondly to use the results of that demonstration to provide a plausible explanation for the variation in the lower value of *x* that has been observed by experiment.

## 2. Possible ordered distributions of trivalent cations in layered double hydroxides   

**Figure 1:**
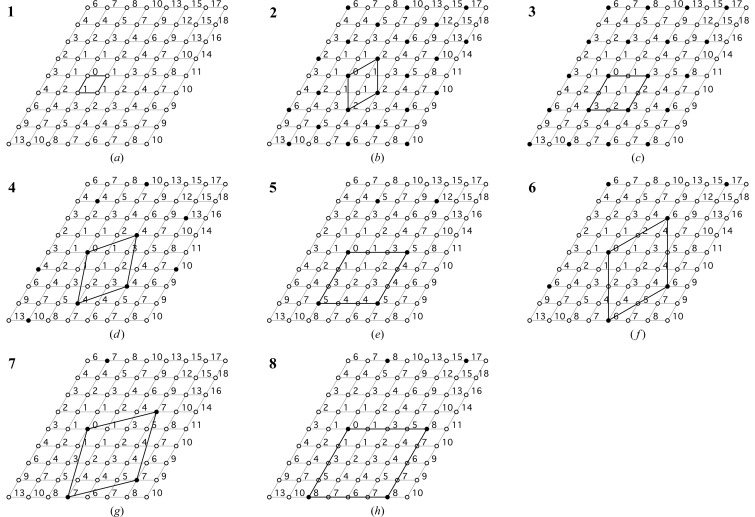
Hexagonal ordered distributions of *M* ^3+^ ions in the octahedral layer of layered double hydroxides. The open circles represent *M* ^2+^ ions and the full circles *M* ^3+^. The full line indicates the supercell. The numbers are explained in the text.

**Figure 2:**
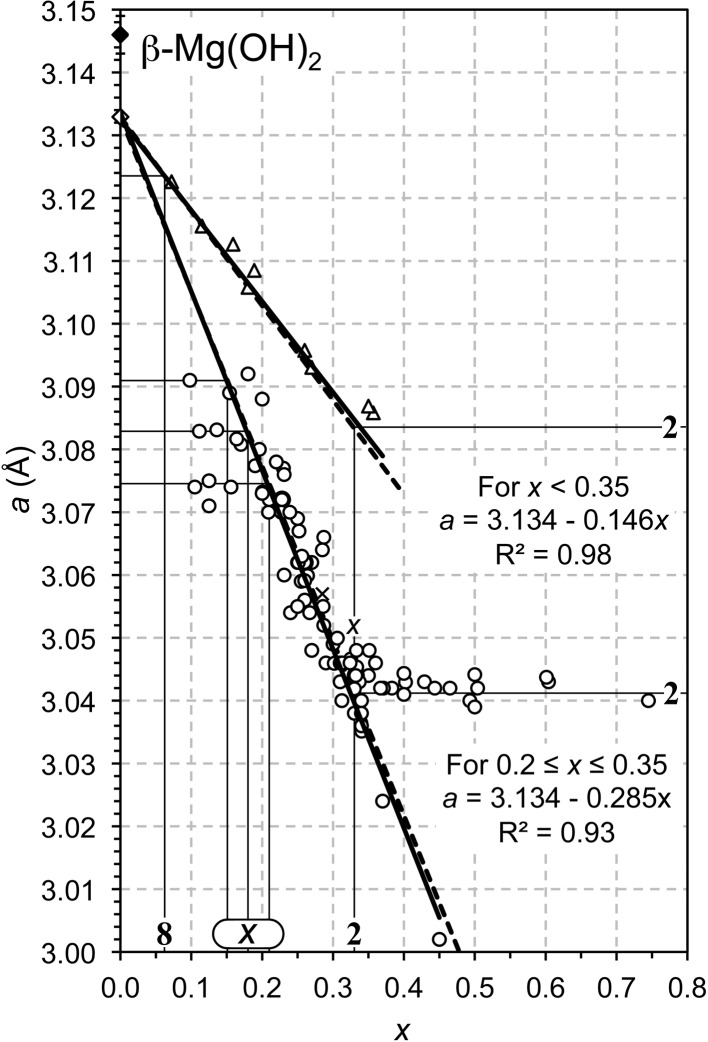
Plot of the *a* parameter against *x* for a range of Mg–Al (circles) and Mg–Ga (triangles) layered double hydroxides (LDH) reported in the literature; the data for the Mg–Al LDH involve a variety of interlayer anions (*i.e.* OH^−^, CO_3_^2−^, NO_3_^−^, Cl^−^) and are from: Mg–Al: Bellotto *et al.*, 1996 ▶; Bîrjega *et al.*, 2005 ▶; Brindley & Kikkawa, 1979 ▶; Budhysutanto *et al.*, 2011 ▶; Gastuche *et al.*, 1967 ▶; Han *et al.*, 1998 ▶; Jinesh *et al.*, 2010 ▶; Kaneyoshi & Jones, 1999 ▶; Kukkadapu *et al.*, 1997 ▶; Mascolo & Marino, 1980 ▶; Miyata, 1980 ▶; Pausch *et al.*, 1986 ▶; Rao *et al.*, 1998 ▶; Sato *et al.*, 1988 ▶; Shen *et al.*, 1994 ▶; Valente *et al.*, 2011 ▶; Xu & Zeng, 2001 ▶; Yun & Pinnavaia, 1995 ▶; Mg–Ga: Bellotto *et al.*, 1996 ▶; López-Salinas *et al.*, 1997 ▶. The full lines are the result of the linear regression analyses of both sets of data and the filled diamond represents the β polymorph of magnesium hydroxide (*i.e.* brucite). The dashed lines represent the values of *a* calculated from theory [using equation (15) in Richardson, 2013*b* ▶]. The open diamond can be taken to represent a theoretical α form of magnesium hydroxide (Richardson, 2013*b* ▶). The bold numbers correspond to the ordered distributions of *M* ^3+^ ions in the octahedral layer that are illustrated in Fig. 1 ▶; the three compositions that are labelled ‘*x*’ are explained in the text.

The hexagonal ordered distributions of *M* ^3+^ ions that correspond to the seven largest values of *x* are shown in Fig. 1 ▶. The open circles in Fig. 1 ▶ represent *M* ^2+^ ions and the full circles *M* ^3+^ ions. For an *M* ^2+^ ion at position 0, the nearest cation neighbours are at position 1, the next-nearest at position 2, followed by 3, 4, 5 *etc.* (*i.e.* the first, second, third, … cation coordination shells). Substitution of *M* ^3+^ for *M* ^2+^ results in a +1 charge and so if the first substitution occurred at position 0, it is often considered that the next substitution would occur no closer than position 2 because of mutual electrostatic repulsions (Brindley & Kikkawa, 1979 ▶; Hofmeister & von Platen, 1992 ▶; Drits & Bookin, 2001 ▶). Continuation of this pattern of substitution gives the arrangement shown in Fig. 1 ▶ (**2**), where each *M* ^3+^ ion is surrounded by 6 *M* ^2+^ ions and the *M* ^2+^:*M* ^3+^ ratio is 2 (and so *x* = 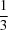); this composition corresponds to the maximum substitution that is observed in the majority of studies of Mg–Al LDH phases, which can be seen by comparing the position of the data points with the thin lines that are labelled with **2** in Fig. 2 ▶, which includes the data from numerous studies that were collated by Richardson (2013*b* ▶) for Mg–Al LDH phases that have a variety of interlayer anions. The full line on Fig. 1 ▶ (**2**) indicates the supercell. In this case the *a* parameter of the supercell is equal to 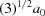, where *a*_0_ is the value for the subcell, where there is no differentiation between cations, *i.e.* as shown in Fig. 1 ▶ (**1**). The actual value of *a*_0_ of course varies with *x*, as shown in the *a*–*x* plots in Richardson (2013*a* ▶,*b* ▶) that include data that were collated from numerous sources (Fig. 1 of Richardson, 2013*a* ▶, for Zn–Al phases; Fig. 2 of Richardson, 2013*a* ▶, for Co–Al phases; Fig. 4*a* of Richardson, 2013*b* ▶, for Ni–Al and Ni–Fe phases; Fig. 6*a* of Richardson, 2013*b* ▶, for Mg–Al and Mg–Ga phases). The next closest ordered distributions are obtained by placing the *M* ^3+^ ions at position 3, which gives the arrangement in Fig. 1 ▶ (**3**), followed by position 4, which gives Fig. 1 ▶ (**4**), and so on. The compositions and values of the supercell *a* parameter are given in Table 1 ▶. Drits & Bookin (2001 ▶) note that for *M* ^2+^:*M* ^3+^ = *Q*, 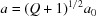. Inspection of Table 1 ▶ shows that this is correct, but also that the values of *Q* are restricted: 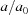 follows the sequence 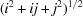, where *i*, *j* = 0, 1, 2, 3, … *etc.* (except for *i* = *j* = 0) and so the possible values of *Q* (*i.e. M* ^2+^:*M* ^3+^ ratios) for ordered distributions of trivalent cations are restricted to the sequence 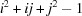; ordered distributions have values of *x* equal to 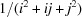 and 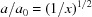. As noted above, Drits & Bookin (2001 ▶) refer to supercell parameters of 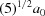 and 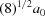; it is evident from Fig. 1 ▶ and Table 1 ▶ that neither of these is possible. The superstructures that are known to exist in the hydrotalcite supergroup are illustrated in a less detailed figure in Mills *et al.* (2012 ▶) who note that the unusually large 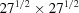 superstructure reported for karchevskyite (by Britvin *et al.*, 2008 ▶) is presumably due to ordering of the interlayer species [because the value of *x* = 0.333 for karchevskyite corresponds to a supercell *a* parameter of 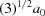; 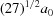 is obtained with *i* = *j* = 3 and so *x* = 0.037].

**Figure 3:**
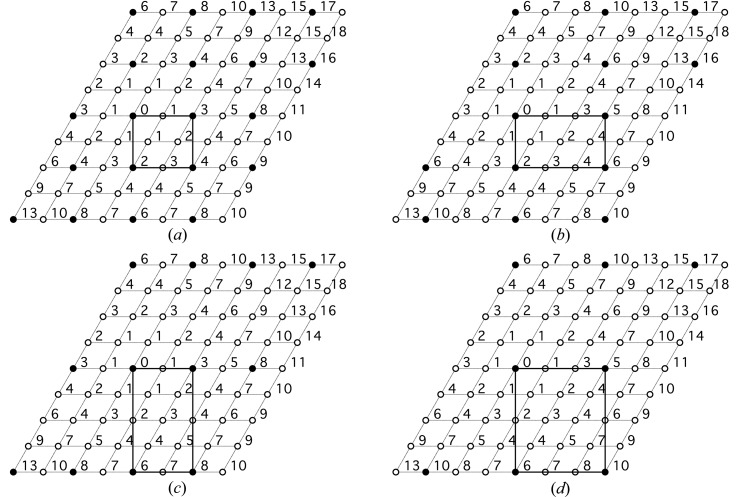
The orthorhombic ordered distributions of *M* ^3+^ ions in the octahedral layer of layered double hydroxides that correspond to the four largest possible values of *x*: (*a*) 0.25; (*b*) 0.167; (*c*) 0.125; (*d*) 0.083. The open circles represent *M* ^2+^ ions and the full circles *M* ^3+^.

Orthorhombic ordered distributions of *M* ^3+^ ions can be created, as illustrated in Fig. 3 ▶ (the open circles again represent *M* ^2+^ ions and the full circles *M* ^3+^). However, it is not obvious why such distributions would occur in preference to the hexagonal distributions that are illustrated in Fig. 1 ▶ because in those cases the trivalent cations are distributed evenly. Nevertheless, Aimoz *et al.* (2012 ▶) claimed recently that the distribution in Fig. 3 ▶(*a*) occurred in a Zn–Al LDH sample that had *M* ^2+^:*M* ^3+^ = 3 because their results were interpreted to indicate that trivalent ions were present in both the second and third metal coordination shells (from Zn), *i.e.* Al^3+^ ions at positions 2 and 3. However, their data do not appear to be conclusive: inspection of their Fig. 9(*d*) shows that whilst there is what they describe as a ‘*local maximum*’ at *s* = 1 (the reader is referred to their paper for the meaning of ‘*s*’), the maximum of the peak envelope is at *s* > 1, which would mean that Al was absent from the third shell, which would support the hexagonal supercell rather than ortho­rhombic.

## 3. The maximum value of *x* and an explanation for the variation in the lower value   

The data collated for Mg–Al LDH systems in Fig. 2 ▶ indicate an interesting phenomenon at low *x*: the minimum value is variable, but it appears to occur at particular fixed values. This observation requires a satisfactory explanation, which can perhaps be obtained by considering the possible ordered distributions of the trivalent ions in the octahedral layer, as detailed above. The range of values of *x* over which Vegard’s Law holds (Vegard, 1921 ▶; West, 1984 ▶; Denton & Ashcroft, 1991 ▶), *i.e.* the extent of solid solution in LDH phases, has been the subject of much discussion. In a seminal paper, Brindley & Kikkawa (1979 ▶) considered that the highest substitution of *M* ^2+^ ions by *M* ^3+^ was near to one in three (*i.e. x* = 0.333), and that the lowest was one in five or six (*x* = 0.200 or 0.167). Their view has been repeated often, particularly in works concerning synthetic Mg–Al preparations, but extensions to the range have been demonstrated regularly, and this is reflected in the data collated from many studies by Richardson for a variety of systems (Richardson, 2013*b* ▶: Mg–Al and Mg–Ga systems, which are reproduced in Fig. 2 ▶; Ni–Al and Ni–Fe; Richardson, 2013*a* ▶: Zn–Al and Co–Al systems). Inspection of Fig. 4 of Richardson (2013*b* ▶) indicates that the upper value of *x* for Ni-based systems appears to be greater than 0.333, perhaps as high as 0.4, and the lower value is less than 0.1. The maximum in the Mg-based systems varies with the method of synthesis, but it is most commonly 0.333, which is clearly evident in Fig. 2 ▶ and in Fig. 6 of Richardson (2013*b* ▶). The *a* and *c*′ parameters for *x* greater than 0.333 are generally essentially constant because those samples consist of a mixture of the LDH phase that has *x* = 0.333 and an Al-rich second phase, which can be crystalline (*e.g.* bayerite) or amorphous. The data points that are included in Fig. 2 ▶ which have values of *a* that continue the linear trend beyond *x* = 0.333 are from Kukkadapu *et al.* (1997 ▶) whose samples involved the terephthalate dianion, C_6_H_4_(COO^−^)_2_, as the charge-compensating interlayer ion.

If the trivalent ions are ordered – albeit with some imperfections that result in the absence of supercell reflections – then the minimum value of *x* that has been observed (to the author’s knowledge) corresponds to distribution **6** in Fig. 1 ▶ for the Ni-based systems, **8** for Mg–Ga (see Fig. 2 ▶), and **4** for Zn–Al. As noted earlier, the minimum value of *x* in the Mg–Al systems is variable and seems to occur at particular fixed values. The steep slope of the linear part of the *a*–*x* plot (Fig. 2 ▶) means that the values of *x* that correspond to where the *a* parameter deviates from linearity are rather exact; they are indicated in the figure by three thin lines labelled ‘*x*’. The line with the lowest value of *x* at approximately 0.15 most likely corresponds to distribution **4**, perhaps with occasional layers of **2** and **3** (a value of *x* = 0.151 would result from ratios for distributions **2**:**4** and **3**:**4** of 1:24 and 1:12, respectively). Whilst it is evident that the other two lines that are labelled ‘*x*’ do not correspond to any of the ordered distributions in Fig. 1 ▶ (the compositions are given in Table 1 ▶), they can in fact be explained by very simple combinations: the middle line is drawn at *x* = 0.1806, which corresponds to a 1:1 mix of distributions **3** and **5**; the right hand line is drawn at *x* = 0.2083, which corresponds to a 1:1 mix of **2** and **6**. Inspection of Fig. 1 ▶ shows that distributions **3** and **5** are related in a straightforward way, as are distributions **2** and **6**. Simple stacking sequences of related ordered distributions of trivalent ions seem therefore to provide a plausible explanation for the compositional trends observed at low *x* in the Mg–Al system.

Whilst compositions with 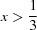 are unusual, they have nevertheless been observed (see Fig. 2 ▶); it is clear from Fig. 1 ▶ that the octahedral layer in such preparations must include trivalent cations that are present in edge-sharing octahedra. Computer simulations have indicated that these could occur as a regular chain structure, with percolation at 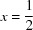 resulting in infinite straight chains of metal–oxygen octahedra containing divalent cations alternating with others containing trivalent cations (Xiao *et al.*, 1999 ▶). Ruby *et al.* (2010 ▶) claim to have produced Fe^2+^–Fe^3+^ LDH phases (*i.e.* the so-called ‘green rust’) that have values of *x* of 0, 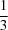, 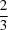 and 1. By analogy with other LDH systems, it seems reasonable to suppose that the phase that has *x* = 0 is an α form of divalent metal hydroxide (Richardson, 2013*a* ▶,*b* ▶). The phases that have *x* > 0 are discussed in detail by Mills *et al.* (2012 ▶), who:

(i) redefine the mineral fougèrite as a Fe^2+^–Fe^3+^ hydroxycarbonate LDH phase that has 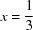;
(ii) define a Fe^2+^–Fe^3+^ oxyhydroxy­carbonate LDH phase that has 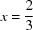 as trébeurdenite (*oxy*hydroxycarbonate because some deprotonation of the main layer hydroxyl groups is proposed);
(iii) name a ferric oxyhydroxy­carbonate LDH phase (*i.e.* 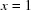) as mössbauerite.

**Figure 4:**
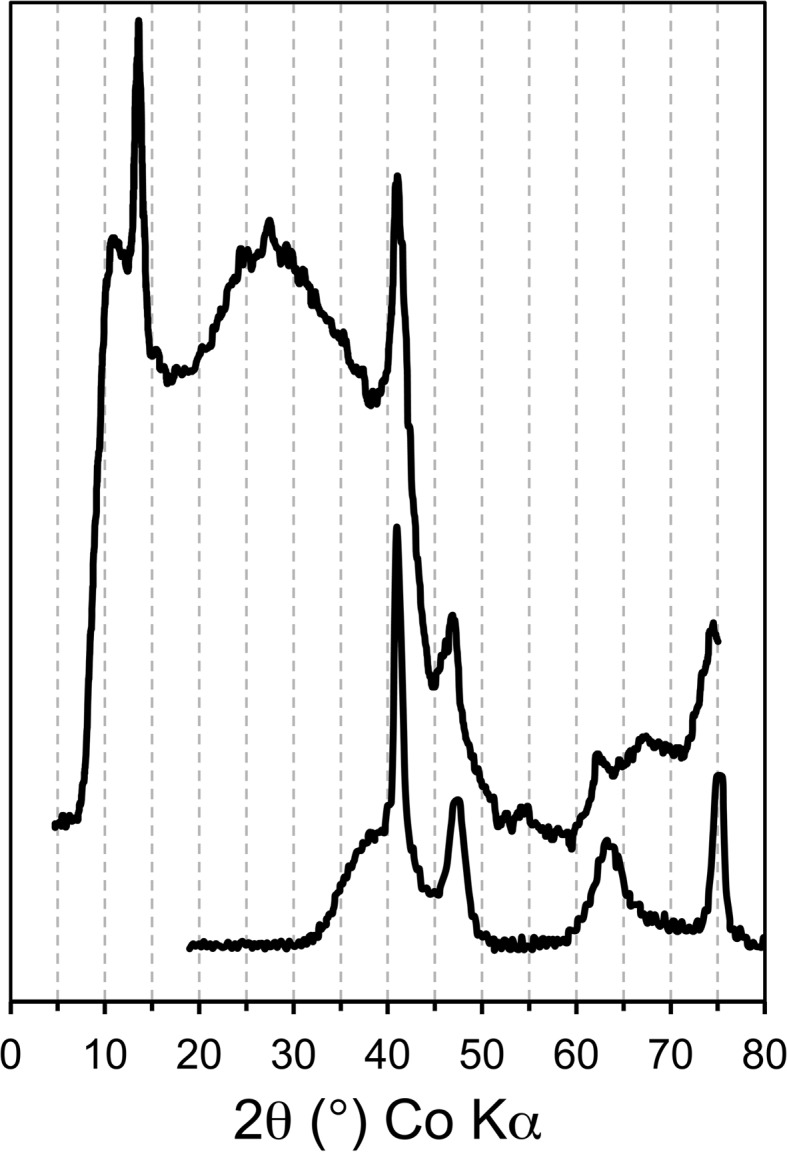
XRD pattern for a Fe^2+^–Fe^3+^ LDH preparation with *x* = 0.83 (upper pattern) compared with a pattern for feroxyhite. The data for feroxyhite were extracted from Fig. 1 ▶ of Drits *et al.* (1993 ▶) and those for the LDH preparation were extracted from Fig. 10*b* of Mills *et al.* (2012 ▶). The pattern for the latter does not appear noisy when compared with the original because it is a plot of the peaks with averaged noise. The data for feroxyhite were converted from Cu *K*α radiation to Co *K*α to facilitate comparison.

Ruby *et al.* (2010 ▶) suggest that a preparation that is made with a composition between any pair of these four phases will consist of a mixture of the two end-members. The quality of the X-ray diffraction (XRD) data presented in Ruby *et al.* (2010 ▶) deteriorates very significantly as the value of *x* increases: they state that ‘*a degradation of the diffractogram is observed with global peak broadening when x increases and some lines are no longer detectable for x values of 0.83 and 1*’. Their XRD data for these two compositions are reproduced with an expanded intensity axis in Fig. 10*b* of Mills *et al.* (2012 ▶), and inspection of those patterns for values of 2θ > 30° reveals a striking similarity with a pattern in Drits *et al.* (1993 ▶) for feroxyhite *i.e.* δ-FeOOH. This is illustrated in Fig. 4 ▶, which compares data that were extracted from Fig. 10*b* of Mills *et al.* (2012 ▶) for the sample that has 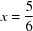 with Drits *et al.*’s pattern for feroxyhite (data converted from Cu *K*α to Co *K*α). The presence of δ-FeOOH is entirely plausible given that it has been observed previously in experiments that used a similar method of sample preparation (Bernal *et al.*, 1959 ▶). The only other large peak on the pattern for 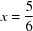 is at about 13.6° 2θ, which corresponds to a *d*-spacing of 7.55 Å (Co *K*α). This is by far the most intense LDH peak on Fig. 10*a* of Mills *et al.* (2012 ▶), and is attributed to the 003 peak of an LDH phase. The next most intense peak (006) would therefore be expected at about 27.43° 2θ (*d* = 3.77 Å) and there is indeed an indication of a peak at this position. Since the other peaks for an LDH phase would be smaller than the 006 peak, it is reasonable to assume that they would be lost in the noise (the original figure must be viewed to appreciate the extent of the noise because Fig. 4 ▶ is a plot of the peaks with averaged noise). The pattern for 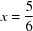 is therefore plausibly explained as being due to a mixture of one LDH phase and feroxyhite, rather than to a mixture of two LDH phases, *i.e.* one with 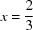 (trébeurdenite) and a second with *x* = 1 (mössbauerite). As a consequence, the validity of mössbauerite seems questionable unless more compelling evidence emerges.

## 4. Summary   

The sequence of hexagonal ordered distributions of trivalent cations that are possible in the main layer of LDH phases has been clarified – including the composition and supercell parameter – and a plausible explanation has been provided for the observed variation in the lower value of *x*.

## Figures and Tables

**Table 1:** The composition and the supercell *a* parameter for the ordered distributions of trivalent cations that are shown in Fig. 1 ▶ *a*_0_ is the value for the cell where there is no differentiation between cations. It is evident that 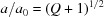 and that it follows the sequence 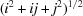, where *i*, *j* = 0, 1, 2, 3, *etc.* (except for *i* = *j* = 0); ordered distributions of trivalent cations therefore have values of *x* equal to 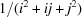 and 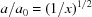.

Position No.	*M* ^2+^/*M* ^3+^ (= *Q*)	*x*	*a* parameter of supercell	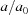	*i*, *j*	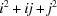
1	0	0	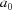	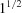	0, 1	1
2	2	0.333	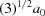	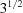	1, 1	3
3	3	0.250	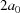	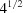	0, 2	4
4	6	0.143	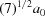	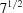	1, 2	7
5	8	0.111	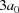	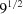	0, 3	9
6	11	0.083	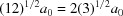	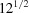	2, 2	12
7	12	0.077	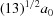	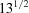	1, 3	13
8	15	0.063	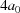	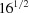	0, 4	16
